# Gene discovery informatics toolkit defines candidate genes for unexplained infertility and prenatal or infantile mortality

**DOI:** 10.1038/s41525-019-0081-z

**Published:** 2019-04-15

**Authors:** Ruebena Dawes, Monkol Lek, Sandra T. Cooper

**Affiliations:** 1 Kids Neuroscience Centre, Kids Research, Children’s Hospital at Westmead, Sydney, NSW 2145 Australia; 2 Discipline of Child and Adolescent Health, Faculty of Health and Medicine, University of Sydney, Sydney, NSW 2006 Australia; 3 Yale School of Medicine, Yale University, 333 Cedar Street, New Haven, CT 06510 USA; 4 The Children’s Medical Research Institute, 214 Hawkesbury Road, Westmead, Sydney, NSW 2145 Australia

## Abstract

Despite a recent surge in novel gene discovery, genetic causes of prenatal-lethal phenotypes remain poorly defined. To advance gene discovery in prenatal-lethal disorders, we created an easy-to-mine database integrating known human phenotypes with inheritance pattern, scores of genetic constraint, and murine and cellular knockout phenotypes—then critically assessed defining features of known prenatal-lethal genes, among 3187 OMIM genes, and relative to 16,009 non-disease genes. While around one-third (39%) of protein-coding genes are essential for murine development, we curate only 3% (624) of human protein-coding genes linked currently to prenatal/infantile lethal disorders. 75% prenatal-lethal genes are linked to developmental lethality in knockout mice, compared to 54% for all OMIM genes and 34% among non-disease genes. Genetic constraint correlates with inheritance pattern (autosomal recessive <<autosomal dominant <X-linked), and is greatest among prenatal-lethal genes. Importantly, >90% of recessive genes show neither missense nor loss-of-function constraint, even for prenatal-lethal genes. Detailed ontology mapping for 624 prenatal-lethal genes shows marked enrichment among dominant genes for nuclear proteins with roles in RNA/DNA biology, with recessive genes enriched in cytoplasmic (mitochondrial) metabolic proteins. We conclude that genes without genetic constraint should not be excluded as potential novel disease genes, and especially for recessive conditions (<10% constrained). Prenatal lethal genes are 5.9-fold more likely to be associated with a lethal murine phenotype than non-disease genes. Cell essential genes are largely a subset of mouse-lethal genes, notably under-represented among known OMIM genes, and strong candidates for gamete/embryo non-viability. We therefore curate 3435 ‘candidate developmental lethal’ human genes: essential for murine development or cellular viability, not yet linked to human disorders, presenting strong candidates for unexplained infertility and prenatal/infantile mortality.

## Introduction

Recent technical advances in high-throughput parallel sequencing technologies is greatly enabling novel gene discovery in Mendelian disorders. However, exome sequencing renders a diagnostic yield of <10% for prenatal phenotypes,^1^ suggesting that many genes critical for human development remain unknown. The traditional paradigm of working from an observed Mendelian phenotype to identify a causative variant and disease gene, is often not a viable option for developmental lethal phenotypes. Thus, determination of the genetic basis for unexplained infertility, recurrent miscarriage, or foetal death, remains an area of great need in clinical genomics.^2^ With many aspects of mammalian embryonic development exquisitely conserved, it is very likely that genes critical for murine development are similarly requisite for human development.

Recent large-scale informatics datasets can provide invaluable tools to help prioritise candidate novel disease genes. For example, the Genome Aggregation Database (gnomAD) v2.1 provides a large reference database of genetic variation identified from exome sequencing for 125,748 individuals, and whole genome sequencing for 15,708 individuals, who do not manifest a paediatric Mendelian condition.^3^ The scale of the dataset enables calculation of ‘observed versus predicted’ genetic variation, and thus scores of genetic constraint to missense or loss-of-function (LoF) variants. Simply put, gnomAD constraint metrics calculate the theoretical frequency of mutation you would expect in each gene based on the mutational frequencies of certain nucleotides and the codon sequence context;^3^ relative to the observed frequency of genetic variation. If a gene is depleted for genetic variants at a population scale, one can infer that there is purifying selection that reduces the incidence of observed variation in this gene. Further studies of regional genetic constraint have applied the same theoretical underpinnings, but on segments within genes, rather than at the whole-gene level—as one means to identify protein sub-domains depleted of mutation.^4–6^

GnomAD analyses revealed that genes intolerant to LoF variants encompass almost all known severe, haploinsufficient human disease genes.^3^ These findings suggested that gnomAD scores of genetic constraint could be a useful tool to prioritise disease genes, and therefore, scores of LoF-constraint have begun to be used by the genomics research community as one means to filter and prioritise candidate disease genes.^7–12^ However, the assumption that genetically constrained genes are more likely to be disease genes has not been evaluated across the breadth of known Mendelian disease genes.

Mouse genome informatics (MGI) provides curated phenotype data for murine models with targeted knock-out of 8433 protein-coding genes.^13^ In addition, the International Mouse Phenotyping Consortium (IMPC) recently published the first stage of an effort to systematically create conditional knock-out (KO) alleles for all protein-coding genes in a murine model, with phenotype data provided for 3820 protein-coding genes in its eighth release.^14,15^

New technologies in genome editing are also being applied in cell biology, with several groups conducting systematic knock-out of coding and non-coding genes among eleven human cell lines.^16–18^ Each group collectively identified a sub-group of genes termed cell-essential genes (essential for viability under cell culture conditions), with good agreement in identified cell ‘essentialomes’ (*n* ~ 1700 genes) between studies. One study also identified cell-line specific essential genes, reflecting discrete modes and capacities of different cell lines to compensate for loss of a given gene; a process termed genetic buffering.^16^

However, it can be challenging for clinical genomics researchers with a long-list of candidate disease variants/genes to cross-reference numerous, informatics-based genome-wide studies on a gene-by-gene basis. Therefore, to aide clinicians and genomics researchers in their hunt for novel disease genes, herein we compile multiple large-scale informatics studies for all human protein coding genes into an easy-to-mine table that integrates: scores of genetic constraint, murine phenotypic information, and the cell ‘essentialome’ (https://github.com/RubyDawes/GD_Informatics_Toolkit/releases/tag/v1.0.0).

Critical assessment of features of 3187 clinically relevant OMIM genes, versus 16,009 protein-coding genes not associated with disease, establish murine phenotypic information is a better raw tool for disease gene prioritisation than scores of genetic constraint; especially for candidate prenatal-lethal genes, with 75% known prenatal-lethal genes linked also to developmental lethal murine phenotypes. Importantly, we curate a list of 3435 ‘candidate developmental lethal’ human genes essential for murine development, or cellular viability, which are not linked currently to human disorders. These genes present plausible candidate genes for apparent infertility or developmental lethality, with detailed ontology mapping further defining subcellular locales and likely molecular functions of putative dominant versus recessive candidate prenatal-lethal genes.

## Results

### Informatics datasets integrated into the Gene Discovery Toolkit

**Fig. 1:**
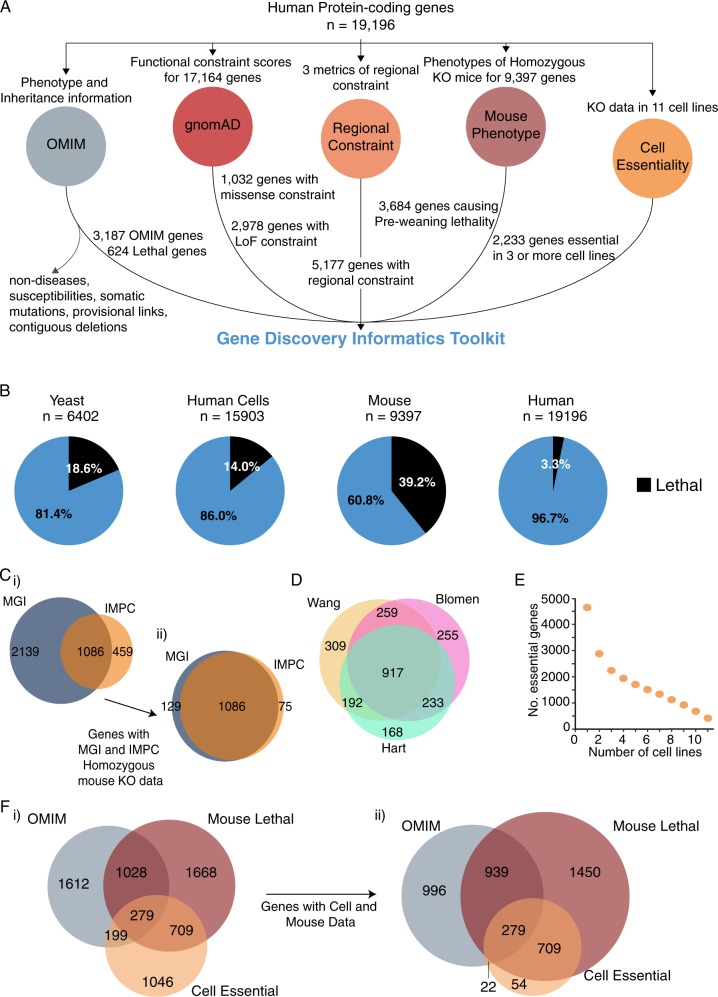
Gene Discovery Informatics Toolkit. **a** Data sources integrated within the Gene Discovery Informatics Toolkit. **b** Proportion of protein-coding genes found to be essential in yeast, human cells, mice and humans. Relative proportions of cell-essential genes are presented relative to the number of genes for which knockouts have been created (see Methods for details). Human-lethal genes were extracted through mining of OMIM database as described in methods, and proportion is shown relative to all protein-coding genes. **c** Venn diagram showing overlap between mouse lethal genes extracted from MGI^13^ and IMPC.^15^ (**i**) Overlap of genes annotated as inducing a pre-weaning lethal phenotype with recessive knockout in MGI and IMPC. (**ii**) 1290 genes with phenotypic information available for homozygous KO in both MGI and IMPC, with 84% concurrence in genes similarly annotated as inducing pre-weaning lethality by both sources. **d** Venn diagram showing overlap between cell ‘essentialomes’ described in the ref. ^16–18^ Analyses include only genes tested in all three studies (15,903/19,169 protein coding genes, see Methods). **e** Number of genes classified as cell-essential among eleven cell lines.^16–18^ Our criteria for an aggregated ‘cell essentialome’ was defined as all genes shown to be essential for cellular viability in three or more cell lines, among any of the three studies. **f** Venn diagrams showing overlap between OMIM genes and mouse lethal and cell essential genes. (**ii**) Overlap of murine and cell essentialomes among all protein coding genes (16,764 genes which have either cell or mouse data, including 3118 OMIM genes shown in the grey Venn circle). (**ii**) Dataset is restricted to include only 8536 genes with both mouse and cell phenotypic information available. Note that areas of circles in Fii are not exactly correlated to numbers of genes but are largely representative of proportions

Genes linked to Mendelian disorders were extracted from OMIM (Online Mendelian Inheritance in Man) and filtered to create a list of 3187 Mendelian disease genes with clinically relevant phenotypes, appended with inheritance information (Fig. 1a, GD Informatics Toolkit Supplementary Table 1; see methods for applied filters). Genes associated with prenatal or infantile lethal human phenotypes were curated manually using an extensive array of phenotypic search terms (see Methods). Clinical phenotypic descriptions were reviewed for each gene to confirm at least one instance where a genetic variant in the gene was deemed to underpin a lethal prenatal or infantile phenotype. Notably, we identified only 624 genes (3% of human protein-coding genes) linked currently to prenatal or infantile lethality identified; in stark contrast to reports that 39% of murine genes are essential for murine development^14^ (Fig. 1b).

The Gene Discovery informatics toolkit integrates murine phenotypic information from MGI,^13^ with the eighth release of IMPC data;^14^ appending extracted information with embryonic, pre-natal, peri-natal, or post-natal lethality (Fig. 1c, GD Informatics Toolkit Supplementary Table 1). Genes defined herein as ‘mouse-lethal’ are those annotated with one or more lethal murine phenotype (MP) terms with recessive knockout from either IMPC or MGI (see Methods). Among 9397 genes for which a recessive murine knock-out model was available, 39.2% result in pre-weaning lethality (<3 weeks of age); with 3684 genes inducing lethality with homozygous knockout (Fig. 1b), and 153 of these linked to lethal phenotypes with either heterozygous or homozygous knock-out (Supplementary Table 1, Sheet 1; see Sheet 4 for 385 additional mouse-lethal genes not captured by recessive null filter). Recessive knockout phenotypes for 1290 genes were available in both IMPC and MGI, with 84% concordance in description of a resultant pre-weaning lethal phenotype (Fig. 1c, ii).

We next integrated ‘cell essentialome’ datasets from three global gene knockout studies, assessing 15903/19196 (83%) of protein coding genes among eleven different cell lines.^16–18^ Each study employed different statistical models to define ‘cell essentialomes’ encompassing ~1580–1878 genes, with overall good agreement between studies (Fig. 1d, e). Only 416 genes were essential in all 11 cell lines (Fig. 1e). Herein we define ‘2233 cell essential genes’ as those genes causing non-viability when knocked out in three or more cell lines.^16–18^ Around 14% of human protein-coding genes are essential for cell viability (aligning well with ~19% genes essential in yeast, see methods) (Fig. 1b). Cell essential genes overlap significantly with murine lethal genes (Fig. 1f), and typically have ancient origins in eukaryotic biology, with conserved roles in fundamental cellular processes such as energy production, DNA/RNA synthesis and replication, protein biosynthesis.^16–18^ Only 21% of cell essential genes (478/2233) are linked currently to human disorders. Cell essential genes not yet linked to human disorders present strong disease candidates for unexplained infertility or early embryonic lethality.

Finally, to each human protein coding gene, we appended gnomAD scores of genetic constraint to missense or loss-of-function (LoF) variants,^3^ as well as aggregated scores for regional missense constraint extracted from three recent studies^4–6^ (see Fig. 1a).

### Genetic constraint is a poor predictor of being a ‘disease gene’—especially for recessive genes

**Fig. 2:**
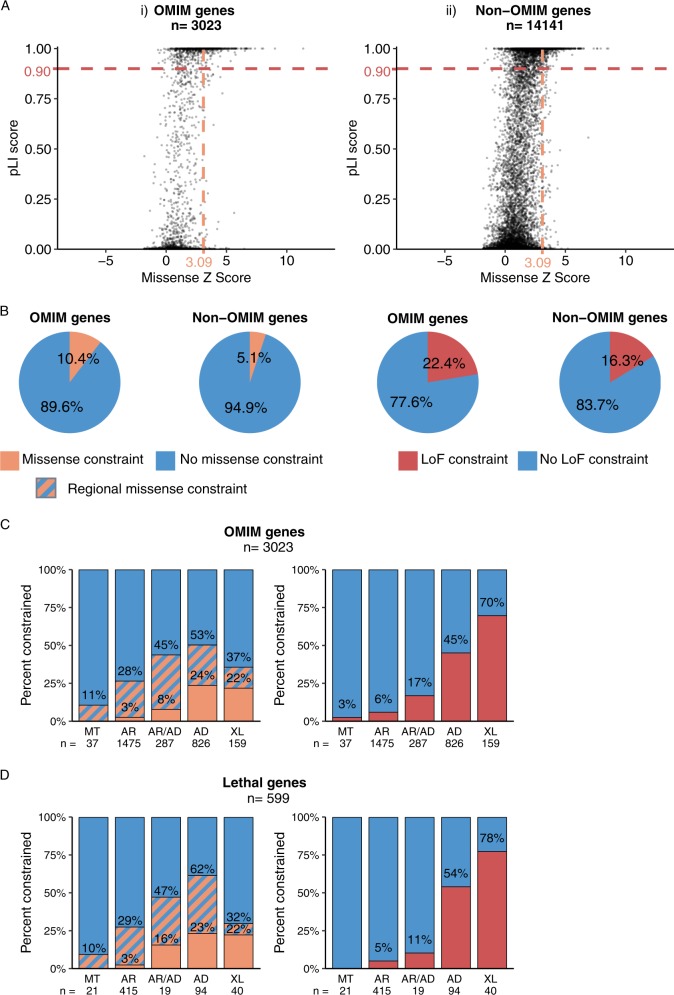
Loss-of-function (LoF) and missense constraint for OMIM versus non-OMIM genes. **a** Scatter plot showing levels of genetic tolerance to LoF (pLI) or missense constraint for 3115 OMIM genes (left) versus 14,757 non-OMIM protein-coding genes (right). Coloured dashed lines indicate thresholds (defined in the ref. ^3^) demarking constraint to missense (mis *z* ≥ 3.09) or LoF (pLI ≥ 0.9) variation. **b** Pie Charts contrasting relative levels of genetic constraint for OMIM versus non-OMIM genes. A significantly higher proportion of OMIM genes than non-OMIM genes show missense constraint (odds ratio OR = 1.68; *p* < 2.2 × 10^−16^) or LoF constraint (OR 1.51; *p* < 2.2 × 10^−16^) using Fisher’s two-sided exact test. **c** Correlation of inheritance pattern with levels of genetic constraint among all OMIM genes: Left: Missense constraint—orange bars are OMIM genes with missense *z* ≥ 3.09. Orange and blue striped bars are OMIM genes with missense *z* < 3.09 and classed as having regional missense constraint by at least one of three metrics described in methods. Right: LoF constraint—red bars are OMIM genes with LoF pLI ≥ 0.9. The number and percentage of OMIM genes in each category are annotated. MT, Mitochondrial; AR, autosomal recessive; AR/AD, autosomal recessive and autosomal dominant; AD, autosomal dominant; XL, X-linked **d** Correlation of inheritance pattern with levels of genetic constraint among 624 curated prenatal-lethal genes (prenatal or infantile mortality)

Analysis of 3187 OMIM genes linked to clinically relevant phenotypes reveals great diversity in scores of genetic constraint (Fig. 2a). Importantly, 75.7% of OMIM genes do not exhibit whole-gene missense or loss-of-function genetic constraint (Fig. 2b, c). To explain in simple terms, genetic variation in these genes broadly mirrors that expected based on random chance, based on tri-nucleotide sequence context and empirically derived mutation rate, as calculated by the algorithm presented in the ref. ^3^ The orange hatched bars in Fig. 2c depict genes determined to show regional genetic constraint, that may reflect functional domains intolerant to genetic variation. Nevertheless, accounting for both whole gene and regional constraint; 57% of known clinically relevant OMIM genes are classified as tolerant to genetic variation (i.e., non-constrained); an unexpected and important finding.

### Levels of genetic constraint correlate with inheritance pattern, with prenatal-lethal genes showing highest levels of genetic constraint

Patterns of missense or loss-of-function (LoF) constraint correlate broadly with whether the inheritance pattern of the associated phenotype is caused by homozygous, heterozygous or hemizygous mutation (Fig. 2c, d). Autosomal recessive (AR) genes rarely demonstrate missense constraint (2.8%) or intolerance to LoF variation (6.1%). In contrast, genes associated with autosomal dominant (AD) disorders show significantly higher levels of missense constraint (23.8%), with 45.3% intolerant to LoF variation. X-linked (XL) genes show intermediate levels of missense constraint (22% XL compared to 2.8% AR and 23.8% AD); though are the most intolerant of LoF variation (69.8%), consistent with hemizygous expression of X-linked genes in males. Our curated list of 624 genes linked to prenatal, perinatal or infantile lethality show the greatest levels of genetic constraint, correlating with inheritance pattern (Fig. 2d AR<<AD<XL).

Highest missense constraint for AD genes may reasonably relate to ‘poison protein’ mode of disease pathogenesis associated with many AD conditions—though it is important to acknowledge AD conditions are linked also to haploinsufficiency, or a combination of both protein dysfunction and protein shortage. Interestingly, among 37/3187 OMIM nuclear-encoded genes annotated as mitochondrial inheritance (MI), none show missense constraint, with only *NDUFS7* showing LoF constraint (34/36 MI genes autosomal recessive; *NDUFA1* and *NDUFB11* are X-linked).

It is also important to emphasise that gnomAD calculation of ‘expected variation’ versus ‘observed variation’ via population-based analytics is tailored to detect genetic constraint of genes due to heterozygous variation, and does not yet factor recessive variation. Scores of genetic constraint therefore inherently hold better inference for autosomal dominant or hemizygous conditions. Thus, genetic constraint is important and should be considered, but genes without genetic constraint should not be excluded as potential novel disease genes—especially for recessive conditions.

### Murine phenotypic information appears a better raw tool for disease gene prioritisation than scores of genetic constraint

**Fig. 3:**
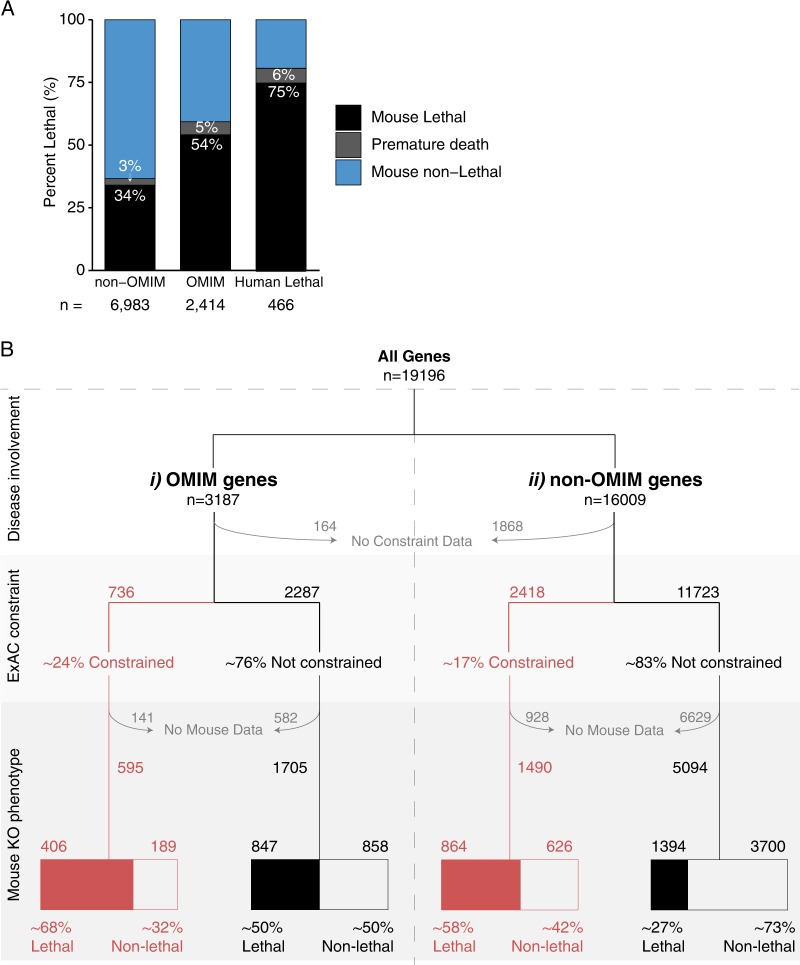
**a** Human-lethal genes (prenatal/infantile lethality) are strongly associated with lethal murine phenotypes. Proportions are based on 466/624 OMIM prenatal/infantile lethality genes for which Murine phenotypic data was available. **b** Flow chart connecting disease involvement with genetic constraint and murine non-viable phenotypes. OMIM genes: constrained genes: 68% linked to a lethal phenotype (murine phenotypic data available for 595/736 constrained genes). Non-constrained genes: 50% linked to a lethal phenotype (murine phenotypic data available for 1705/2287 non-constrained genes). Non-OMIM genes: constrained genes: 58% linked to a lethal phenotype (murine phenotypic data available for 1490/2418 constrained genes). Non-constrained genes: 27% were linked to a lethal phenotype (murine phenotypic data available for 5094/11723 non-constrained genes). Murine non-viability is more prevalent among constrained genes (68% among OMIM constrained genes and 58% among non-OMIM constrained genes). However, many non-constrained genes are nevertheless associated with non-viable murine phenotypes (27% among OMIM genes without constraint and 50% among non-OMIM genes without constraint)

Fifty-four percent of OMIM genes are linked to early lethality (developmental or neonatal) in knockout mice, compared to 34% of non-disease genes (Fig. 3a). Importantly, among our curated list of 624 genes associated with human prenatal, perinatal or infantile lethality—75% were also associated with pre-weaning lethality in a murine model (Fig. 3a), with an additional 6% of prenatal-lethal genes classified as inducing murine premature death. Thus, our attention focusses intently on 2377 genes known essential for murine development, not yet linked to human disease (Fig. 3b and see Figure 1f, ii).

### Cell essential genes present strong candidates for unexplained infertility or early embryonic lethality

Cross referencing cell-essential genes with murine-essential genes, we noted 709 cell essential genes not known to be associated with human disorders, though linked to embryological lethality in mice (see Fig. 1f). We believe these genes are plausible candidates for non-viability of gametes or early embryos in humans. Unexpectedly, 76 genes classified as cell-essential were not linked to murine lethal phenotypes (GD Informatics Toolkit Supplementary Table 1, sheet 2); however 5/76 genes were associated with sub-viable murine phenotypes and 39/76 with phenotypic abnormality of a certain cell type or organ (annotated as ‘abnormal cellular phenotype’).

### Ontology mapping defines distinct subcellular localisations and functional roles between dominant and recessive prenatal-lethal genes

**Fig. 4:**
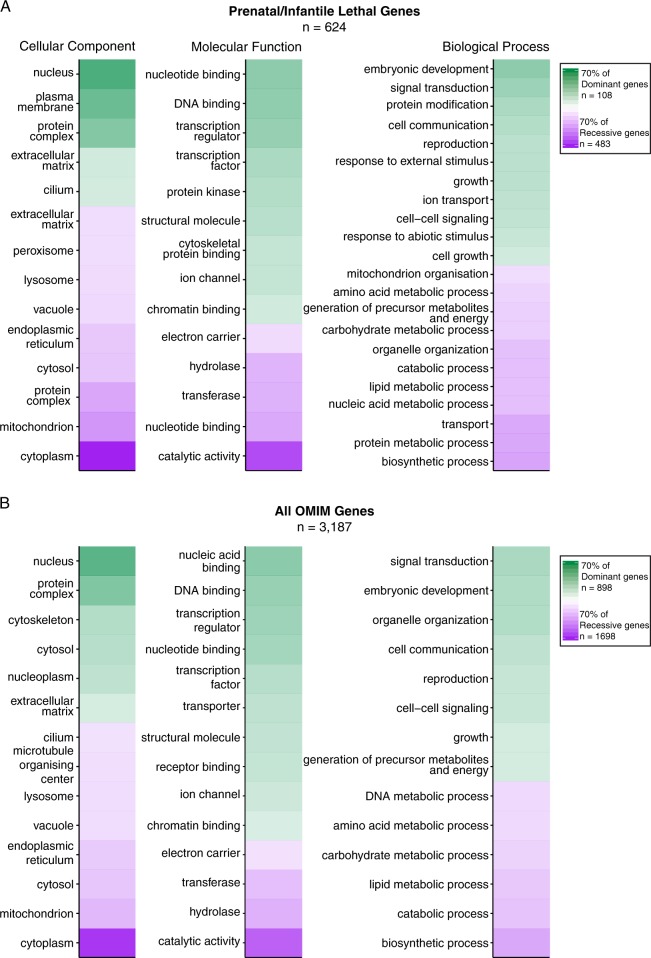
Ontology analysis of 624 known prenatal-lethal genes, compared with all 3187 OMIM genes. **a** Comparative analysis of gene ontology between dominant (AD or X-linked dominant) and recessive (AR or X-linked recessive) prenatal-lethal genes. Gene ontology terms for each gene were compiled, then GO terms comparatively enriched in either dominant or recessive gene families were determined (subcellular localisation, molecular function and biological processes). Comparative metrics were exported from cytoscape with a *p*-value cutoff of 0.025 with data visualisation performed in R. Green: GO terms significantly comparatively enriched among Dominant lethal genes, with intensity of colour correlating with the proportion of genes in category annotated with that term. Purple: GO terms significantly comparatively enriched terms among Recessive lethal genes. **b** Comparative analysis of gene ontology between dominant and recessive among all 3187 OMIM genes. Ontology analyses is available in https://github.com/RubyDawes/GD_Informatics_Toolkit/releases/tag/v1.0.0. Highly general terms are excluded from the figure (cellular component, cell, binding etc.). Some ontology terms are abbreviated for readability. Only the 14 most significantly overrepresented categories are visualised

Ontological analyses of 624 known prenatal-lethal genes, stratified by dominant (AD or X-linked dominant) versus recessive (AR or X-linked recessive) inheritance, defines striking segregation in subcellular localisation and molecular functions (Fig. 4a). Dominant prenatal-lethal genes are highly enriched for protein products with nuclear localisation, molecular roles in DNA binding and transcription factor activity and biological processes such as gene regulation, differentiation, embryonic development and signal transduction. In contrast, protein products of recessive prenatal-lethal genes more typically reside within cytoplasmic organelles (particularly mitochondria), with molecular roles as metabolic or biosynthetic enzymes (or regulators) (Fig. 4a). These trends held true for analysis of all 3187 OMIM genes stratified into dominant or recessive genes (Fig. 4b).

### 3435 plausible candidates for non-viable human developmental phenotypes; potentially relevant to infertility or recurrent death in utero

Critical analyses of ‘essentialomes’ for murine or cellular viability led us to consider deeply non-OMIM genes within these gene lists. Genes essential for murine or cellular viability are strong candidates for human developmental lethal phenotypes. This assumption is supported strongly by critique of 624 known prenatal-lethal genes, with 75% associated also with a developmental lethal murine phenotype (Fig. 3a). We therefore collate a curated list of 3435 ‘candidate developmental lethal’ human genes determined essential for murine or cellular viability, but not yet linked to human disease (GD Informatics Supplementary Table 1; sheet 3).

**Fig. 5:**
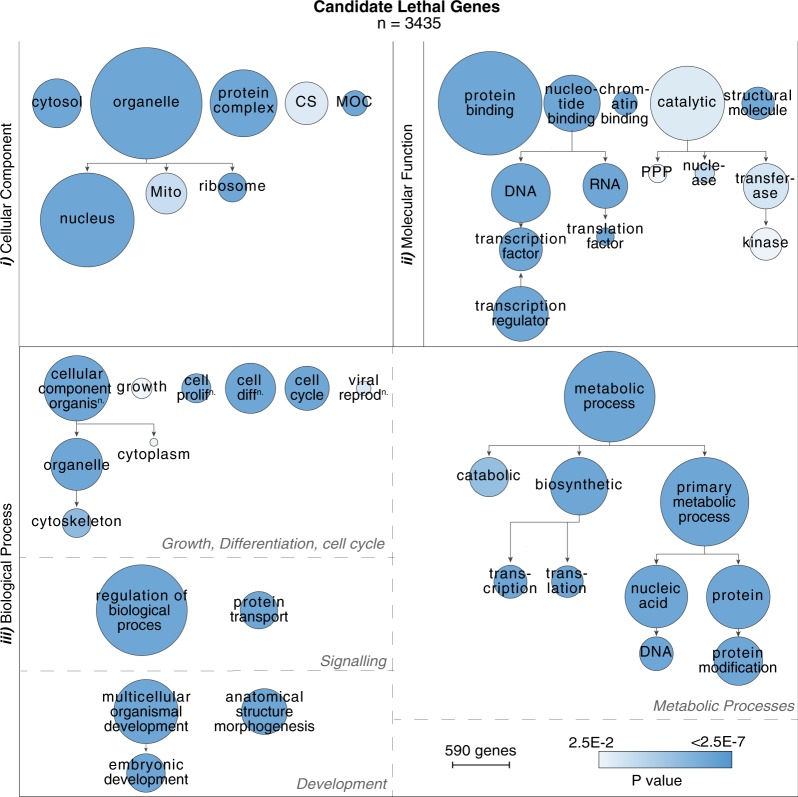
Ontology analysis of 3435 candidate prenatal/infantile lethal genes. Gene Ontology categories enriched among 3435 candidate prenatal/infantile lethal genes compared with all 19,196 protein-coding genes as a background were visualised using BiNGO cytoscape plugin. Size of circles is proportional to the number of genes in the ontology category and colour is proportional to the *p*-value of enrichment of this GO term. Layout was manually organised for ease of interpretation, with biological process enriched terms separated into four categories: (1) Growth, differentiation, cell cycle; (2) Signalling; (3) Development and (4) Metabolic processes. Some ontology terms are abbreviated for readability

Gene ontology analyses of the 3435 ‘candidate developmental lethal’ genes shows a striking enrichment for nuclear genes (Fig. 5i) with profound enrichment of genes linked to DNA and RNA binding and transcriptional regulation (Fig. 5, ii, 1496/3435); highlighting a void in our current understanding of complex transcriptional regulation of human genes required for successful embryogenesis. These genes were more likely to show whole gene genetic constraint (610/1445, 42.2%); and thus present good candidates as putative autosomal dominant or X-linked candidate disease genes. In comparison, mitochondrial genes among ‘candidate developmental lethal’ genes (Fig. 5i; 282/3435 genes) were more frequently non-constrained at the whole gene level (44/275 genes, 16%). Thus, these genes present good candidates for recessive conditions.

### Odds ratio analysis

**Fig. 6:**
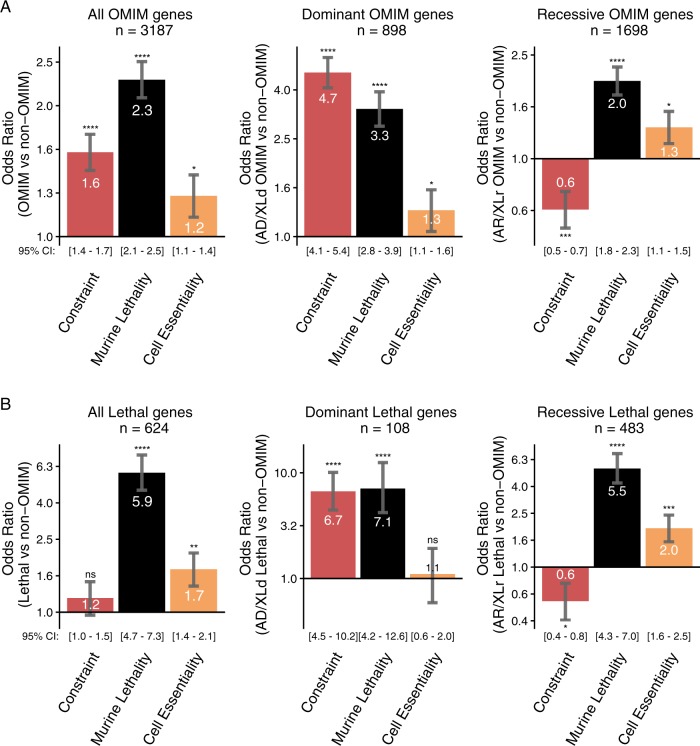
Odds ratio (OR) analyses synthesising relevance of scores of genetic cosntraint and model organism phenotypic data, with likelihood of being a disease gene. **a** All OMIM genes. **b** 624 Human-lethal disease genes. Red bars: murine lethality. Black bars: genetic constraint. Orange bars: cell essentiality. Odds-ratio statistical significance determined via Fishers two-sided exact test. Numbers in brackets represent the 95% confidence interval for each value. Maximum statistical significance determined in R is *p* < 2.2 × 10^−16^. Thus: ****p < 2.2 × 10^−16^; ***p < 5.0 × 10^−7^; **p < 5.0 × 10^−5^; *p < 5.0 × 10^−2^

To synthesise our overall findings, odds-ratio analyses reveal manifestation of a severe animal phenotype is most strongly correlated with being a disease gene (Fig. 6). Compared to non-disease genes, OMIM genes are 2.29 fold more likely to be associated with developmental murine lethality in a recessive knock-out mouse model (Fig. 6a, left, black bar), with prenatal-lethal genes 5.9-fold more likely (Fig. 6b, left, black bar). Whereas compared to non-disease genes, OMIM genes are only 1.56 times more likely to show whole-gene constraint (Fig. 6a, left, red bar).

Stratification of dominant versus recessive genes highlights the relevance of scores of genetic constraint; relative to non-disease genes, dominant genes are 4.68-fold enriched for genes with missense or loss-of-function constraint (Fig. 6a, middle, red bar), and 6.7-fold enriched among prenatal-lethal genes (Fig. 6b, middle, red bar).

Cell essential genes showed the least predictive power (Fig. 6a, b, orange bar). However, we believe cell-essential genes are extremely strong candidates for non-viability of gametes or early embryos; thus complicating phenotypic detection, diminishing the representation of these genes among known disease genes.

## Discussion

To guide novel gene discovery, and particularly in clinical fields of infertility and developmental lethality, herein we compile multiple large-scale informatics studies for all human protein-coding genes. Systematic analyses of 3187 known disease genes have yielded core lessons to inform novel gene discovery in rare Mendelian disorders.

(1) Levels of genetic constraint correlate broadly with inheritance pattern; dominant OMIM genes show greater constraint than recessive genes, X-linked genes show greatest loss-of-function constraint. However, the majority of known OMIM disease genes show tolerance to genetic variation. Therefore, genes without genetic constraint should not be excluded as potential novel disease genes, and especially for recessive conditions (<10% constrained). (2) Murine lethality with recessive knock-out is very strongly associated with OMIM genes linked to the most severe of human phenotypes (prenatal, neonatal or infantile lethality). 75% of 624 known prenatal-lethal genes are linked to developmental lethal phenotypes in mice, with an additional 6% of prenatal-lethal genes linked to premature murine death. (3) Cell essential genes are largely a subset of murine developmental lethal genes, and plausibly are so necessary for cellular viability that early human embryos succumb and are never detected. (4) We compile a list of 3435 candidate developmental lethal genes that include all human genes linked to developmental lethality in murine models, or cellular non-viability (with recessive knockout), not yet known to be associated with human genetic conditions; presenting strong candidate genes for apparent infertility, recurrent miscarriage, foetal death, or disorders involving early infantile lethality.

Our study could not assess effectively non-protein coding genes, due largely to non-availability of mouse phenotyping data, and a significant gap in our biological understanding of the roles of non-coding genes for gene ontology studies. However non-protein coding genes are recognised increasingly as causes of Mendelian disease,^19,20^ and are also subject to purifying selection and genetic constraint.^21^

While inclusion of ‘undiscovered disease genes’ within our dataset of non-disease genes represents an unavoidable caveat; critical assessment of 624 genes linked to prenatal/infantile lethality among 3187 OMIM genes confirms strong relevance of a developmental lethal murine phenotype to inform undiscovered prenatal-lethal genes. We further establish that dominant prenatal-lethal genes show high levels of genetic constraint and are enriched greatly for nuclear proteins with roles in RNA/DNA biology, whereas recessive prenatal-lethal genes are commonly non-constrained and enriched for catabolic and biosynthetic enzymes or regulators.

Interestingly, 25% of genes causing severe lethal phenotypes in humans were not recapitulated in recessive KO mice (Fig. 3a). One explanation for this discordance is that murine recessive knock-out models only complete absence of a gene product, and does not capture pathogenetic mechanisms due to missense mutations or truncating variants causing gain-of-function, partial dysfunction of gene products; or a mixture of both in recessive disease. With many gene products among known prenatal-lethal genes linked to differentiation, signalling and embryogenesis; further explanations may relate to differences between humans and mice in gene regulation during embryonic development.

In summary, we elucidate key principles for balanced consideration of scores of genetic constraint, mode of inheritance, model organism phenotypic information, and disease relevance of known mechanistic insight for the candidate gene, to help prioritise novel disease gene candidates. We hope dissemination of our candidate developmental lethal gene list, as well as communication of insights gained from our curation and critical interrogation of all known prenatal-lethal genes, will advance gene discovery efforts in this challenging subset of human disorders.

## Methods

### Obtaining a list of genes with clinically relevant phenotypes from OMIM

A list of OMIM genes was downloaded from OMIM (https://www.omim.org/downloads/) with license on 2018-03-10. Genes were filtered to create a list of Mendelian disease genes with clinically relevant phenotypes, and appended with all associated phenotypes and their inheritance patterns. Inclusion criteria: (1) Genes listed among 19,196 protein-coding genes defined by Human Gene Nomenclature Committee (HGNC); (2) Genes for which the molecular basis of the phenotype is known. Exclusion criteria: (1) Phenotypes annotated as non-diseases or susceptibilities; (2) Phenotypes caused by somatic mutations; (3) Genes only provisionally linked to a phenotype; (4) Genes linked to contiguous genomic deletions associated with disease phenotype.

### Extracting and curating a list of genes linked to pre/perinatal lethality

The OMIM Application Programming Interface (API) was used to query text fields across the entire database for terms associated with lethality either before birth or shortly after birth. Search terms are listed in https://github.com/RubyDawes/GD_Informatics_Toolkit/releases/tag/v1.0.0; Supplementary Table 2, sheet 1 with raw hits detailed in Supplementary Table 2, sheet 2. The clinical phenotypic descriptions for each gene were reviewed, excluding any gene where there was no explicit evidence of a monogenic variant within the gene associated with prenatal, perinatal or infantile lethality. The inheritance pattern(s) linked to lethal phenotype(s) is defined within Column 11 Supplementary Table 1, Sheet 1. During manual curation we split genes into two lists: List A contains 344 genes associated with time of death stated explicitly as before birth or before 3 months of age. List B includes these genes, as well as any genes containing with the age of death imprecisely defined, though with clear inference to within the infancy period (i.e., ‘death in infancy’, ‘shortly after birth’, etc.). Six hundred and twenty-four genes in total met these slightly less stringent criteria. List A and B were analysed separately—and when shown to share similar properties, were combined to improve statistical strength of our analyses.

### Deriving a cell essential gene list

‘Cell essentialome’ datasets were derived from three recent studies of genome-wide screens for cell-essential genes in human cell lines.^16–18^ Data on essentiality in each of the 11 cell lines throughout the three studies were extracted from the Supplemental information and collated. Cell essential hits in each cell lines and tallies available in Supplementary Table 2, sheet 3. We classified ‘cell essential’ as showing a requirement for cellular viability with recessive knock-out in three or more cells lines.

Links to Supplemental information extracted:

Blomen: www.sciencemag.org/content/350/6264/1092/suppl/DC1

Wang: www.sciencemag.org/content/350/6264/1096/suppl/DC1

Hart: https://www.cell.com/fulltext/S0092–8674(15)01495–6#secsectitle0130

### Extracting mouse knockout phenotype data

Mouse Genome Informatics Data was downloaded from http://www.informatics.jax.org/downloads/reports/index.html and appended with information on low-level Mammalian Phenotype (MP) terms, high-level MP terms and alleles. This information was used to mine for all phenotypes associated with homozygous KO alleles throughout the genome. Only alleles that affected one gene and annotated as null/knockout or hypomorph were included. The 8th release of IMPC mouse phenotype data was downloaded from the IMPC ftp site (ftp://ftp.ebi.ac.uk/pub/databases/impc/) and filtered to include only MP terms associated with homozygous KO mice. A unified list of ‘mouse lethal’ genes was identified through manual curation querying MP terms associated with phenotypic lethality among the joint MGI and IMPC dataset (lethal MP terms queried are defined within Supplementary Table 2, sheet 4). 3684 genes confirmed to be annotated with one or more lethal MP term(s) with recessive knock-out were thus classified as ‘mouse lethal’ genes for statistical comparisons presented within Figs. 1–5, with 153/3684 genes linked to lethal phenotypes with either heterozygous or homozygous knock-out. Details of all additional genes (*n* = 385) associated with lethal murine phenotypes, not captured by our ‘recessive null’ filter, are provided within GD Informatics Toolkit Supplementary Table 1, Sheet 4; including lethal phenotypes due to heterozygous knockout, or lethality due to a genetic variant other than a null allele.

### Extracting gnomAD constraint scores

Scores of genetic constraint derived from gnomAD release 2.1 for all human protein-coding genes were downloaded from gnomAD downloads page (http://gnomad.broadinstitute.org/downloads). A gene was classified as having whole-gene constraint if scores met the cutoff recommended in the ref. ^3^ (≥3.09 for missense constraint, ≥0.9 for LoF constraint).

### Collating regional constraint scores

An aggregated list of genes with regional constraint were derived and collated from three studies.^4–6^ (1) Genes with regional differences in missense constraint were downloaded from,^5^ who classified genes with regional missense constraint as those with a fraction of expected versus observed genetic variation of (γ) ≤ 0.6.^5^ (2) Genes defined as those with regional constraint within coding regions were downloaded from the github repository associated with^4^ (https://github.com/quinlan-lab/ccrhtml), who classified genes with regional missense constraint as those with coding region constraint within the 99th percentile among all protein-coding genes.^4^ (3) 1996 genes depleted for truncating variants in regions predicted to avoid nonsense mediated decay was downloaded from the ref. ^6^

### Integrating cell-essential genes, murine knockout phenotypic data and scores of genetic constraint into an easy-to-mine informatics toolkit

All data was collated into one spreadsheet containing all protein-coding genes in HGNC annotated with OMIM information, cell essentiality data, mouse phenotype data for mouse orthologs of human genes, as well as gnomAD and regional constraint scores. All analyses were conducted in R using this integrated database. All code and data is available at https://github.com/RubyDawes/GD_Informatics_Toolkit/releases/tag/v1.0.0, which will be updated iteratively within the github repository upon release of new source data.

### Gene ontology (GO) analysis

GO analysis was performed using the Biological networks gene ontology tool (BiNGO) cytoscape plug-in.^22^ Generic GO Slim was used for all analyses. Overrepresentation of ontology categories in gene groups was calculated with a significance cutoff of 0.025 using a Hypergeometric test with Benjamini and Hochberg false discovery rate (FDR) correction. List 19,196 human protein-coding genes was used as the background set. Results were imported into R for data visualisation.

### Extracting yeast essential genes

Yeast essential genes were extracted from the *Saccharomyces* genome database (SGD) (https://www.yeastgenome.org).^23^ All genes associated with Yeast Phenotype Ontology (YPO) terms ‘viable’ and ‘inviable’ were downloaded from the YPO page in the database (https://www.yeastgenome.org/ontology/phenotype/ypo) and filtered to include only null mutations.

### Reporting Summary

Further information on experimental design is available in the Nature Research Reporting Summary linked to this article.

## Supplementary information

Reporting Summary

Supplementary Table 1

Supplementary Table 2

## Data Availability

All source data, custom scripts, and datasets generated during the current study have been made available at https://github.com/RubyDawes/GD_Informatics_Toolkit. As well as being directly available in the above repository, download links for all source data have been provided in the methods.

## Supplementary information

**Supplementary information** accompanies the paper on the *npj Genomic Medicine* website (10.1038/s41525-019-0081-z).

## Code availability

All code used to complete analysis during the current study is available in the GitHub repository, https://github.com/RubyDawes/GD_Informatics_Toolkit.

## References

[1] Vora NL, Hui L (2018). Next-generation sequencing and prenatal ‘omics: advanced diagnostics and new insights into human development. Genet. Med..

[2] Filges I, Friedman JM (2015). Exome sequencing for gene discovery in lethal fetal disorders—harnessing the value of extreme phenotypes. Prenat. Diagn..

[3] Lek M (2016). Analysis of protein-coding genetic variation in 60,706 humans. Nature.

[4] Havrilla JM (2019). A map of constrained coding regions in the human genome. Nat. Genet..

[5] Samocha, K. E. et al. Regional missense constraint improves variant deleteriousness prediction. https://www.biorxiv.org/content/10.1101/148353v1 (2017).

[6] Coban-Akdemir Z (2018). Identifying genes whose mutant transcripts cause dominant disease traits by potential gain-of-function alleles. Am. J. Hum. Genet..

[7] Rodan LH (2016). A novel neurodevelopmental disorder associated with compound heterozygous variants in the huntingtin gene. Eur. J. Hum. Genet..

[8] Kosmicki JA (2017). Refining the role of de novo protein-truncating variants in neurodevelopmental disorders by using population reference samples. Nat. Genet..

[9] Jin SC (2017). Contribution of rare inherited and de novo variants in 2871 congenital heart disease probands. Nat. Genet..

[10] Moutton S (2018). Truncating variants of the DLG4 gene are responsible for intellectual disability with marfanoid features. Clin. Genet..

[11] Singh, T. et al. Rare schizophrenia risk variants are enriched in genes shared with neurodevelopmental disorders. https://www.biorxiv.org/content/10.1101/069344v1 (2016).

[12] Alhuzimi E (2018). Properties of human genes guided by their enrichment in rare and common variants. Hum. Mutat..

[13] Smith CL (2018). Mouse Genome Database (MGD)-2018: knowledgebase for the laboratory mouse. Nucleic Acids Res..

[14] Dickinson ME (2016). High-throughput discovery of novel developmental phenotypes. Nature.

[15] Koscielny G (2014). The International Mouse Phenotyping Consortium Web Portal, a unified point of access for knockout mice and related phenotyping data. Nucleic Acids Res..

[16] Blomen VA (2015). Gene essentiality and synthetic lethality in haploid human cells. Science.

[17] Wang T (2015). Identification and characterization of essential genes in the human genome. Science.

[18] Hart T (2015). High-resolution CRISPR screens reveal fitness genes and genotype-specific cancer liabilities. Cell.

[19] Zhang F, Lupski JR (2015). Non-coding genetic variants in human disease. Hum. Mol. Genet..

[20] Makrythanasis P, Antonarakis S (2013). Pathogenic variants in non-protein-coding sequences. Clin. Genet..

[21] Petrovski S (2015). The intolerance of regulatory sequence to genetic variation predicts gene dosage sensitivity. PLoS Genet..

[22] Maere S, Heymans K, Kuiper M (2005). BiNGO: a Cytoscape plugin to assess overrepresentation of gene ontology categories in biological networks. Bioinformatics.

[23] Cherry JM (2012). Saccharomyces Genome Database: the genomics resource of budding yeast. Nucleic Acids Res..

